# Risk Factors for Mosquito House Entry in the Lao PDR

**DOI:** 10.1371/journal.pone.0062769

**Published:** 2013-05-20

**Authors:** Alexandra Hiscox, Phasouk Khammanithong, Surinder Kaul, Pany Sananikhom, Ruedi Luthi, Nigel Hill, Paul T. Brey, Steve W. Lindsay

**Affiliations:** 1 Institut Pasteur du Laos, Vientiane, Lao PDR; 2 London School of Hygiene and Tropical Medicine, London, United Kingdom; 3 Khammouane Provincial Health Office, Thakhek, Lao PDR; 4 Nam Theun 2 Power Company, Vientiane, Lao PDR; 5 School of Biological and Biomedical Sciences, Durham University, Durham, United Kingdom; Université Pierre et Marie Curie, France

## Abstract

**Background:**

Construction of the Nam Theun 2 hydroelectric project and flooding of a 450 km^2^ area of mountain plateau in south-central Lao PDR resulted in the resettlement of 6,300 people to newly built homes. We examined whether new houses would have altered risk of house entry by mosquitoes compared with traditional homes built from poorer construction materials.

**Methodology/Principal Findings:**

Surveys were carried out in the Nam Theun 2 resettlement area and a nearby traditional rice farming area in 2010. Mosquitoes were sampled in bedrooms using CDC light traps in 96 resettlement houses and 96 traditional houses and potential risk factors for mosquito house entry were recorded. Risk of mosquito house entry was more than twice as high in traditional bamboo houses compared with those newly constructed from wood (Putative Japanese Encephalitis (JE) vector incidence rate ratio (IRR) = 2.26, 95% CI 1.38–3.70, P = 0.001; Anopheline IRR = 2.35, 95% CI: 1.30–4.23, P = 0.005). Anophelines were more common in homes with cattle compared against those without (IRR = 2.32, 95% CI: 1.29–4.17, P = 0.005).Wood smoke from cooking fires located under the house or indoors was found to be protective against house entry by both groups of mosquito, compared with cooking in a separate room beside the house (Putative JE vector IRR = 0.43, 95% CI: 0.26–0.73, P = 0.002; Anopheline IRR = 0.22, 95% CI: 0.10–0.51, P<0.001).

**Conclusions/Significance:**

Construction of modern wooden homes should help reduce human-mosquito contact in the Lao PDR. Reduced mosquito contact rates could lead to reduced transmission of diseases such as JE and malaria. Cattle ownership was associated with increased anopheline house entry, so zooprophylaxis for malaria control is not recommended in this area. Whilst wood smoke was protective against putative JE vector and anopheline house entry we do not recommend indoor cooking since smoke inhalation can enhance respiratory disease.

## Introduction

Mosquito house entry can be reduced through simple changes in house design, such as closing eaves, installing a ceiling, screening external doors and windows and a general improvement in quality of construction materials [1]. In these instances house entry rates are probably reduced by physically blocking or decreasing the number of holes through which a mosquito may gain access to a home. Houses can also be made less suitable for indoor resting mosquitoes by making them well lit, with few places for adult vectors to rest, and this is often cited as one of the reasons for the decline in malaria in Europe [2]. Raising houses on stilts can also reduce mosquito house entry [3], [4] by interfering with host-seeking behaviour.

Anecdotal evidence has suggested that the smoke created by burning biomass fuels inside houses may repel host-seeking mosquitoes, although an in-depth literature review [5] found little evidence that smoke from fires led to a corresponding reduction in malaria.

Limiting exposure to mosquito bites should reduce the risk of exposure to infections such as Japanese encephalitis (JE) and malaria. For example, a recent randomised controlled trial of house screening in The Gambia showed that installing screened ceilings or full screening of houses with fly-screened doors and windows, and closing the eaves resulted in a 50% decline in the risk of anaemia due to malaria, a major killer of young children [6]. In Sri Lanka the incidence of malaria among residents of poor-quality housing was up to 2.5 fold higher compared with a population living in improved housing [7]. Although one study investigating risk factors for culicine mosquitoes in The Gambia found that closed eaves reduced the risk of house entry for this genus [8] a subsequent intervention study showed that culicines entered houses through doors and not the eaves [9]. Little is known about whether improvements in house construction can reduce mosquito house entry rates in other parts of the world, including South-East Asia.

The Nam Theun 2 (NT2) hydroelectric project in south-central Lao PDR, is one of the largest recent development projects in South-East Asia. The project is predicted to generate an average income of US$80 million per year, with Lao government revenues expected to reach a total US$2 billion over the period of a 25 year concession agreement. Hydropower is generated by the force of water released from a reservoir measuring 450 km^2^ in area, descending 348 m to a power station. Flooding of a mountain plateau to create this reservoir resulted in the resettlement of 1,310 households and 6,300 people, into 16 villages settled along the southern shore line of the reservoir. Families were provided with a newly constructed wooden house built to considerably higher standards than traditional houses in the area. Preliminary studies carried out in 312 randomly selected houses in the resettlement area during 2009 indicated that resettlement style houses were at lower risk of house entry by putative JE vectors than a small sample of traditional houses (N = 15) and that houses located in more agricultural parts of the resettlement area were at increased risk of entry by this group of mosquitoes [10].

The specific objectives of the present study were to determine household-level risk factors for mosquito house entry in areas affected by the NT2 hydroelectric project and to relate these risk factors to improvements in housing design which could be incorporated into future development programmes. We sampled mosquitoes from the bedrooms of an equal number of traditional and resettlement-style houses located in the NT2 resettlement area, as well as in a neighbouring area located downstream of the reservoir, and were able to identify household-level risk factors for house entry by potential JE and malaria vectors.

## Materials and Methods

### Ethics Statement

Before commencing sampling in each village a meeting was held with the village head to explain the purposes of this work and to address any questions. Informed oral consent was given by the head of each household after explaining the study in the local language and answering any questions. The study was approved by the Lao Ministry of Health and the ethics committee of the London School of Hygiene and Tropical Medicine. Local approval was granted by the district health offices of Nakai and Gnommalath and by the Health Programme Management Unit of NTPC.

### Study Area

The study was carried out between August and October 2010 in resettled villages and in villages downstream of the reservoir in Khammouane Province, south-central Lao PDR. Mosquito sampling took place in equal numbers of resettlement and traditional-style houses in every study village. The climate in the area is tropical with one hot, rainy season between May and October of each year. During the study period the total rainfall was 1,393 mm and average temperature 25.4°C.

**Figure 1:**
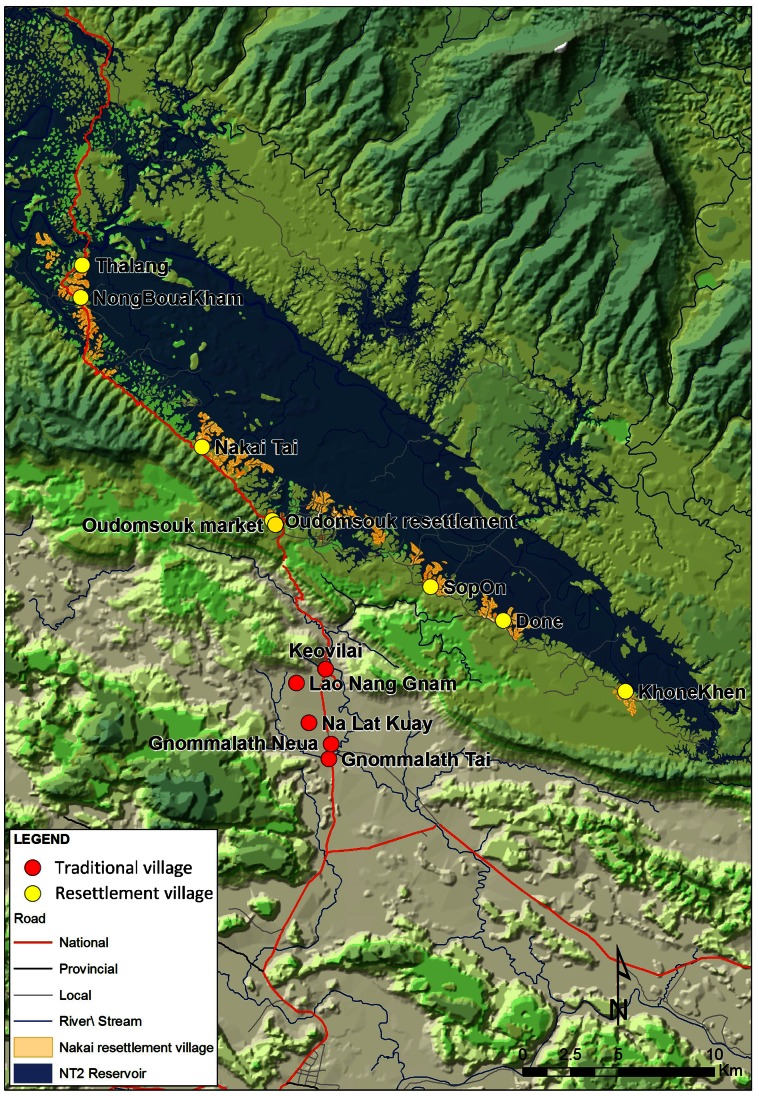
Map to show the location of villages in which sampling took place. The locations of traditional villages are shown in red, resettlement villages in yellow.

Mosquito sampling took place in 8 villages of Nakai district which were distributed along the southern shore of the reservoir and had been resettled as part of the NT2 resettlement programme. From north to south (see Figure 1) these villages were: Thalang (17°50′10.6″N, 105°02′59.9″E, ), NongBouaKham (17°49′15.8″N, 105°02′57.3″E), Nakai Tai (17°45′04.3″N, 105°06′32.8″E), Oudomsouk resettlement (17°42′57.2″N, 105°08′35.7″E), Oudomsouk market (17°42′59.2″N, 105°08′51.6″E), SopOn (17°41′04.5″N, 105°13′16.4″E), Done (17°40′07.1″N, 105°15′24.2″E) and KhoneKhen (17°38′06.5″N, 105°09′34.6″E). These villages were 540–551 m above sea level. Local people had been relocated during the dry season of 2007/2008, just prior to reservoir inundation. Whilst resettlement-style housing dominated in these villages, traditional houses had also been constructed within the villages by people migrating to the area or by families wishing to expand their living space. Villages were separated from the forest by a dirt road and an area of cleared land. At full impoundment level the reservoir reached within about 15 m of some houses on the periphery of the village and hilly, agricultural land formed the remainder of the land cover.

Study houses were also selected from 5 villages in Gnommalath district, downstream of the reservoir: Gnommalath Neua (17°36′37.6″N, 105°10′19.5″E), Gnommalath Tai (17°36′11.7″N, 105°10′0.58″E), Keovilai (17°38′36.2″N, 105°10′09.1″), Lao Nang Gnam (17°38′17.4″N, 105°09′34.6″E) and Na Lat Kuay (17°36′39.5″N, 105°09′40.0″E). These settlements were between 164–185 m above sea level and were mostly surrounded by wet rice agriculture. Houses in these villages were built mainly in a traditional manner with some resettlement-style houses. Some families were resettled due to construction of a downstream water channel but other families had decided to renovate their homes, choosing to build them from hard wood in a style similar to that used in the resettlement programme. All villages were located within 1 km of a tarmac road and had all-weather access to this road.

In both areas the primary occupations of local people included farming, fishing, animal husbandry (cows, buffaloes, pigs, goats, chickens and ducks) and shop keeping.

### Household Selection

Equal numbers of traditional and resettlement study households were selected through a process of simple random sampling, after numbering and categorizing all houses in the study villages as traditional or resettlement style. In the resettlement area maps of each village were available which included plot numbers for each area of land on which a house was constructed and these plot numbers used for selection. In the traditional villages sketch maps were made of each village showing the location of traditional and resettlement houses. These locations were numbered consecutively and these numbers used for randomization. Based on our earlier findings, it was estimated that sampling 96 traditional and 96 resettlement houses would allow detection of a risk factor that doubled the risk of mosquito house entry, with 80% power at the 5% significance level (Epi Info Version 3.5.3, Centers for Disease Control and Prevention. 2011. Atlanta).

### Entomology

Mosquitoes were collected in bedrooms using CDC light traps (John Hock company, Gainsville, Florida). Traps were positioned with the bulb 150 cm above the floor, approximately 50 cm from the foot end of an occupied insecticide-treated bed net with two adults sleeping inside (B-52 Golden Horse Brand, Netto Manufacturing Co. Ltd., Thailand). Houses were sampled between 1800 h and 0800 h six nights a week with each house sampled on one occasion only. Mosquitoes were returned to the field laboratory in cool boxes, killed by freezing at −20°C for at least 20 minutes and identified morphologically using keys to the mosquitoes of Thailand [11], [12].

### Risk Factor Surveys

For each study house the following potential risk factors for mosquito house entry were recorded: village, type of house (traditional or resettlement), style of veranda (open or closed), construction materials (wood or other – mainly bamboo sheeting), roofing material (corrugated iron or other – majority thatch or wooden tiles), condition of external doors and windows (well-fitting resettlement-style shutters, other covering, or open), location of the kitchen (room at the side of the house, separate building, under house or in main living area), number of insecticide-treated bednets, other methods of vector control used by the householders, height of house above the ground, house length, house depth, distance to the nearest toilet, presence of a ceiling, number of people sleeping in the house, television ownership, electricity supply, animal ownership and whether large animals were kept under or around the house at night.

### Statistical Methods

Data from light traps which were not functioning upon collection were excluded from further analysis. The following variables were excluded from the risk factor analysis because they occurred in less than 1% of households: presence of a ceiling, closed eaves, screened windows and doors, use of mosquito coils, burning repellent plants, use of insecticidal spray and indoor residual spraying.

Univariate analyses were performed for each individual risk factor using a generalized linear model with a negative binomial distribution and log link function. Following univariate analysis each risk factor with P<0.1 was incorporated into a multivariable model which was refined through a process of backwards stepwise elimination using a likelihood ratio test. Where P<0.1 for the likelihood ratio test the variable was deemed to contribute significantly to the model and remained in the final multivariable model.

## Results

Surveys took place in 96 traditional and 96 resettlement-style houses between August and October 2010. One hundred and fourteen study houses were located in the Nakai resettlement area with the remainder located in traditional villages, downstream of the reservoir, in Gnommalath district.

**Figure 2:**
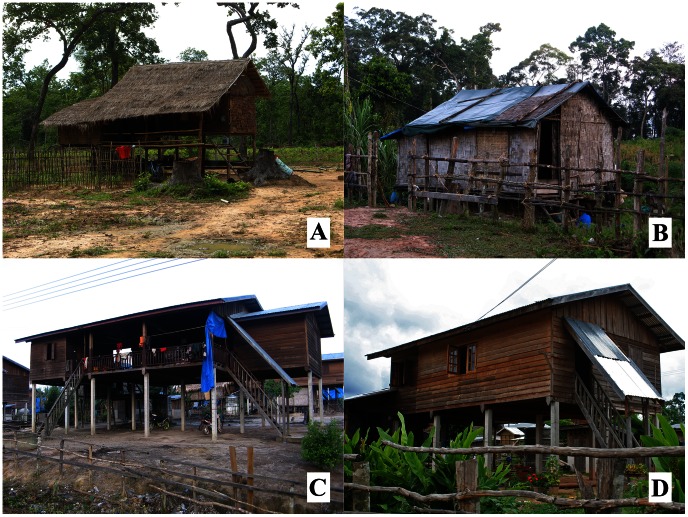
Typical resettlement and traditional houses in the study area. Traditional houses (A and B) are normally constructed from bamboo thatch with roofs made from thatch, wooden tiles or corrugated iron. Doors and windows are poorly fitting and there are many gaps in the walls and floors. Traditional houses are raised on stilts on average 1.27 m above the ground. Resettlement houses in contrast (C and D) are built from pre-dried hard wood with corrugated iron roofs. They generally have fewer gaps in the walls and floors then a traditional house and have well-fitting doors and windows. They are raised on stilts an average of 2.66 m above the ground. The houses shown in this figure are for illustrative purposes and may not have been sampled during the study.

Resettlement and traditional houses differed markedly in their design and construction (Figure 2). Resettlement houses were elevated on stilts higher above the ground (stilts 2.66 m high, 95% CI: 2.45–2.86 m) than traditional houses (stilts 1.27 m high, 95% CI: 1.16–1.37 m). Resettlement houses were constructed from high quality hard wood with few gaps in the walls and floors, as well as tightly fitting windows and doors. Resettlement roofs were made from corrugated iron and the internal area of a house was larger (mean 86.3 m^2^, 95% CI: 77.8–94.8 m^2^) than a traditional house (mean 57.2 m^2^ 95% CI: 51.8–62.6 m^2^). Traditional homes were constructed from bamboo, had many gaps in the walls and floors, had poorly fitted windows and doors and the roofs were made from thatch, wooden tiles or corrugated iron.

A total of 1,797 mosquitoes (1,500 females) were collected, of which 39.2% were *Anopheles philippinensis* and 15.1% were *Culex tritaeniorhynchus*. Other recorded species were: *An. nivipes* (8.6%), *Cx. whitmorei* (7.9%), *An. aconitus* (6%), *Cx. bitaeniorhynchus* (4%), *An. peditaeniatus* (3.5%), *Cx. vishnui* (3.1%), *Cx. quinquefasciatus* (2.8%), *Cx. fuscocephala* (1.5%) and *An. vagus* (1.3%). Other species, including *Aedes albopictus*, were present at low densities (fewer than 20 individuals sampled).

Subsequent analyses were performed by pooling mosquitoes into two groups. Suspected JE vectors were, in order of abundance: *Cx. tritaeniorhynchus, Cx. whitmorei, Cx. bitaeniorhynchus, Cx. vishnui, Cx. quinquefasciatus, Cx. fuscocephala* and *Cx. gelidus*. Potential malaria vectors included all anophelines, in order of abundance: *An. philippinensis, An. nivipes, An. aconitus, An. peditaeniatus, An. vagus, An. barbirostris, An. annularis* and *An. tessellatus*. Identification of risk factors for anophelines was performed for houses only in the downstream villages since only 5 specimens were collected from resettlement villages. Analysis for putative JE vectors was carried out combining data from the two areas, with “area” included in the multivariable model to account for spatial differences.

Univariate analyses (see Table 1) revealed twelve variables that were significantly associated with the outcome measures for putative JE vectors, these were: area (downstream traditional or resettlement), village, house construction material, veranda style, condition of doors and windows, location of kitchen, ITNs per person, untreated nets present or absent, cow ownership, buffalo ownership and any large animals or buffaloes kept below the house. Significant outcome measures for anophelines in univariate analyses were: village, house construction material, veranda style, location of kitchen and cow ownership (see Table 2).

**Table 1:** Risk factors for putative vectors of Japanese encephalitis caught in houses.

		Univariate model		Multivariable model	
	N	IRR (95% CI)	P-value	IRR (95% CI)	P-value
**Village type**					
Resettlement villages	114	1.00		1.00	
Traditional downstream villages	78	4.94 (3.47–7.04)	<0.001	4.00 (2.71–5.89)	<0.001
**Village**					
Thalang	22	1.00			
NongBouaKham	6	47.67 (8.73–260.41)	<0.001		
Nakai Tai	20	12.1 (2.52–58.11)	0.002		
Oudomsouk resettlement	18	15.89 (3.31–76.17)	0.001		
Oudomsouk market	4	13.75 (1.95–97.18)	0.009		
SopOn	18	15.28 (3.18–73.38)	0.001		
Done	18	4.89 (0.92–25.97)	0.063		
KhoneKhen	8	5.5 (0.84–36.06)	0.076		
Lao Nang Gnam	16	47.44 (10.11–222.68)	<0.001		
Keovilai	22	53 (11.61–241.99)	<0.001		
Na Lat Kuay	22	69.5 (15.26–316.43)	<0.001		
Gnommalath Tai	6	18.33 (3.13–107.23)	0.001		
Gnommalath Neua	12	68.75 (14.29–330.64)	<0.001		
**Type of house**					
Traditional	96	1.00			
Resettlement	96	0.93 (0.67–1.29)	0.664		
**House construction materials**					
Wood	126	1.00		1.00	
Other	66	1.95 (1.38–2.75)	<0.001	2.26 (1.38–3.70)	0.001
**Roofing materials**					
Corrugated iron	183	1.00			
Other	9	0.90 (0.41–2.00)	0.803		
**Veranda style**					
Open	115	1.00			
Closed	77	0.54 (0.38–0.77)	0.001		
**External doors and windows**					
Resettlement style shutters	80	1.00		1.00	
Other covering	96	1.69 (1.19–2.39)	0.003	0.88 (0.53–1.45)	0.615
None (open)	16	0.18 (0.07–0.47)	0.001	0.17 (0.06–0.48)	0.001
**Height on stilts (cm)**	192	1.00 (1.00–1.00)	0.247		
**House area (m^2^)**	192	1.00 (0.99–1.00)	0.107		
**Distance to the nearest toilet (cm)**	192	1.00 (1.00–1.00)	0.860		
**Number of people sleeping in the house**	192	1.00 (0.90–1.12)	0.969		
**Television ownership**					
No television	45	1.00			
Owns television	147	1.33 (0.89–1.98)	0.168		
**Where does cooking take place?**					
At the side of the house	103	1.00		1.00	
Completely separate building/no kitchen	45	1.90 (1.28–2.83)	0.001	1.14 (0.73–1.79)	0.571
Underneath house/main living area	44	0.44 (0.27–0.70)	0.001	0.43 (0.26–0.73)	0.002
**ITNs per person**	192	0.55 (0.31–0.96)	0.035		
**Untreated nets**					
Absent	159	1.00			
Present	33	2.01 (1.32–3.05)	0.001		
**Own animals**					
No animals	32	1.00			
Any animals	160	1.14 (0.73–1.79)	0.561		
No chickens	84	1.00			
Chickens	108	1.14 (0.81–1.59)	0.452		
No ducks	98	1.00			
Ducks	94	1.24 (0.89–1.73)	0.201		
No pigs	153	1.00			
Pigs	39	1.15 (0.76–1.72)	0.508		
No cows	156	1.00			
Cows	36	2.05 (1.36–3.08)	0.001		
No buffaloes	163	1.00			
Buffaloes	29	1.93 (1.24–3.00)	0.004		
**Large animals kept below the house**					
No large animals underneath	146	1.00			
Any large animals underneath	46	1.78 (1.27–2.49)	0.001		
No pigs under	165	1.00			
Pigs under	27	1.38 (0.87–2.19)	0.178		
No cows under	174	1.00			
Cows under	18	1.39 (0.80–2.41)	0.241		
No buffaloes under	181	1.00			
Buffaloes under	11	2.37 (1.22–4.59)	0.011		

IRR = Incidence rate ratio. N is the number of households at each level.

**Table 2:** Risk factors for anopheline mosquitoes caught in houses of traditional downstream villages.

		Univariate model		Multivariable model	
	N	IRR (95% CI)	P-value	IRR (95% CI)	P-value
**Month of collection**	78				
September	38	1.00			
October	40	1.37 (0.86–2.17)	0.186		
**Village**	78				
Lao Nang Gnam	16	1.00		1.00	
Keovilai	22	1.24 (0.63–2.44)	0.527	1.03 (0.51–2.07)	0.928
Na Lat Kuay	22	2.13 (1.09–4.16)	0.027	1.68 (0.77–3.64)	0.190
Gnommalath Tai	6	0.70 (0.26–1.91)	0.490	0.57 (0.20–1.65)	0.297
Gnommalath Neua	12	0.24 (0.10–0.57)	0.001	0.35 (0.13–0.92)	0.034
**Type of house**	78				
Traditional	39	1.00			
Resettlement	39	0.72 (0.45–1.14)	0.160		
**House construction materials**	78				
Wood	47	1.00		1.00	
Other	31	1.83 (1.14–2.93)	0.012	2.35 (1.30–4.23)	0.005
**Roofing materials**	78				
Corrugated iron	74	1.00			
Other	4	0.49 (0.16–1.44)	0.193		
**Veranda style**	78				
Open	61	1.00		1.00	
Closed	17	0.32 (0.18–0.58)	<0.001	0.51 (0.23–1.11)	0.089
**External doors and windows**	78				
Resettlement style shutters	27	1.00			
Other covering	48	1.23 (0.75–2.01)	0.411		
None (open)	3	0.87 (0.25–3.12)	0.851		
**Height on stilts (cm)**	78	1.00 (0.99–1.00)	0.584		
**House area (m^2^)**	78	0.99 (0.99–1.00)	0.274		
**Distance to the nearest toilet (cm)**	78	1.00 (1.00–1.00)	0.620		
**Number of people sleeping in the house**	78	1.00 (0.86–1.15)	0.972		
**Television ownership**	78				
No television	16	1.00			
Owns television	62	1.30 (0.73–2.32)	0.372		
**Where does cooking take place?**	78				
At the side of the house	36	1.00		1.00	
Completely separate building/no kitchen	27	1.04 (0.62–1.75)	0.869	1.10 (0.63–1.92)	0.730
Underneath house/main living area	15	0.14 (0.07–0.28)	<0.001	0.22 (0.10–0.51)	<0.001
**ITNs per person**	78	0.74 (0.36–1.55)	0.427		
**Untreated nets**	78				
Absent	53	1.00			
Present	25	1.29 (0.79–2.11)	0.317		
**Own animals**					
No animals	11	1.00			
Any animals	67	1.10 (0.56–2.14)	0.782		
No chickens	26	1.00			
Chickens	52	0.83 (0.51–1.36)	0.460		
No ducks	33	1.00			
Ducks	45	1.19 (0.74–1.89)	0.477		
No pigs	57	1.00			
Pigs	21	0.95 (0.56–1.60)	0.842		
No cows	51	1.00		1.00	
Cows	27	1.92 (1.18–3.12)	0.008	2.32 (1.29–4.17)	0.005
No buffaloes	65	1.00			
Buffaloes	13	1.57 (0.85–2.90)	0.151		
**Large animals kept below the house**					
No large animals underneath	51	1.00			
Any large animals underneath	27	1.18 (0.73–1.92)	0.505		
No pigs under	65	1.00			
Pigs under	13	1.00 (0.54–1.86)	1.000		
No cows under	64	1.00			
Cows under	14	1.43 (0.78–2.59)	0.245		
No buffaloes under	71	1.00			
Buffaloes under	7	1.38 (0.62–3.08)	0.428		

IRR = Incidence rate ratio. N is the number of households at each level.

Since the dominant land cover (hilly farmland and reservoir in the resettlement area and flooded ricefields in the downstream area) was uniform for each group of villages, and that confidence intervals for village-level risk were wide, area was ultimately preferred as the measure of geographical variation used in the multivariable model for putative JE vectors.

After accounting for confounders, houses in the traditional downstream area were at 4 times greater risk of suspected JE vector entry compared with houses in the resettlement villages (Incidence rate ratio (IRR) = 4.00, 95% CI: 2.71–5.89, P<0.001). Houses made from bamboo or other non-wooden materials were at 2.26 times greater risk of house entry by this group of mosquitoes than houses with wooden floors and walls (IRR = 2.26, 95% CI: 1.38–3.70, P = 0.001). In contrast, houses with open windows and doors had an 83% reduced risk of putative JE vectors in bedrooms compared with houses which had well-fitting wooden doors and window shutters (IRR = 0.17, 95% CI: 0.06–0.48, P = 0.001). The presence of a cooking fire located in the main living area or directly underneath the house was associated with a 57% reduced risk of suspected JE vector house entry compared with houses in which the fire was located in a separate room at the side of the house (IRR = 0.43, 95% CI: 0.26–0.73, P = 0.002).

In the traditional downstream area anopheline mosquitoes were abundant and the risk of house entry did not differ greatly between villages. In only one village, Gnommalath Neua, was there a reduced risk of anopheline house entry compared with the baseline comparison village of Lao Nang Gnam (IRR = 0.35, 95% CI: 0.13–0.92, P = 0.034). As the dominant land cover, size of villages and population did not seem to vary between downstream villages, Lao Nang Gnam was arbitrarily allocated as the baseline comparison village.

Similar to the results for putative JE vectors, risk of house entry by anophelines which may transmit malaria was discovered to be greater in houses which were constructed from bamboo thatch and other non-wooden materials compared with wooden houses (IRR = 2.35, 95% CI: 1.30–4.23, P = 0.005). Burning a fire in the main living area or directly below the house was associated with a 78% reduced risk of anopheline house entry compared with cooking in a separate room at the side of the house (IRR = 0.22, 95% CI: 0.10–0.51, P<0.001). Ownership of a cow more than doubled the risk of anopheline house entry compared with houses not owning a cow (IRR = 2.32, 95% CI: 1.29–4.17, P = 0.005).

## Discussion

Our analysis of risk factors for house entry by putative vectors of JE and malaria shows that in the NT2 resettlement area and in a traditional rice farming area immediately downstream of the reservoir, the type of housing and how people use their house affects the risk of mosquitoes entering bedrooms and presumably the risk of mosquitoes biting residents of these houses. Many of the mosquito species collected during the course of this study have previously been incriminated as vectors of JE or malaria, but it was not feasible to conduct incrimination studies within the scope of this study. Additional studies to measure mosquito infection status and compare the health of people living in traditional vs. improved housing would enable associations to be made between the impacts of reduced mosquito house entry and transmission of JE virus and malaria.

Overall, houses of all construction types which were located in the traditional rice farming area downstream of the reservoir, were at greater risk of putative JE vector entry compared with houses in the resettlement area, and anophelines were much more abundant in these villages. Rice fields form one of the primary habitats for vectors of both JE and malaria in Asia [13], [14], [15] and proximity to mosquito breeding sites is a previously-documented risk factor for exposure to mosquitoes and mosquito-borne disease in a variety of settings [16], [17], [18], [19]. In Gnommalath Neua village, malaria vectors were less commonly trapped than in other villages of the traditional area and this might be explained by the village being slightly closer to the road with comparatively fewer surrounding rice fields than the index village, Lao Nang Gnam.

Altitude was consistent between villages in the same area, but there was an approximately 370 m difference in elevation above sea level between the resettlement and the traditional area which might have contributed to differences in mosquito densities and risk of house entry. A study in North-eastern Tanzania reported declining anopheline vector densities, and reduced malaria entomological inoculation rates correlating with increasing altitude in four villages between 860 m and 1,565 m above sea level, although relatively few houses were sampled in each village and catch sizes varied widely [20]. The effect of altitude on mosquito density and species composition is largely unknown in South-East Asia, particularly over small-scale geographical areas. As differences in altitude are often associated with changes in climate, topography and land use, it may be difficult to assess which aspects are of most importance in explaining differences in mosquito populations.

Despite differences between the two areas, improved houses located in both resettled and downstream villages were found to have reduced rates of mosquito house entry compared with traditional houses located in both areas. The finding that improvements in housing design reduce exposure to mosquitoes is supported by previous studies [1], [4], [6], [8], [21], [22], [23], [24], [25]. In this area building houses from straight-edged wooden slats probably reduces the number of gaps in the walls and floors of a house through which a mosquito might enter, compared with poorly constructed bamboo housing which is likely to have many more holes through which mosquitoes could enter and out of which host odours could pass.

Surprisingly, the risk of house entry by putative JE vectors was reduced by 83% in houses with open doors and windows compared with houses which had well-fitting wooden shutters. This result is in stark contrast with the conventional wisdom that closing or screening doors and windows reduces mosquito house entry [6]. There are at least three explanations for this finding; (1) host odours may have been less concentrated in houses with completely open doors and windows, thus attracting fewer mosquitoes, (2) the result is a sampling artefact, since a mosquito in a closed room may fly for longer, searching for an exit, thereby increasing its probability of being collected in a light trap, compared with open houses and (3) this result occurred by chance.

Where cooking took place directly underneath the house or in the main living area, house entry by putative JE vectors and anopheline mosquitoes was reduced by 57% and 78% respectively, compared with houses in which cooking took place in a separate room.

The literature relating smoke from domestic fires to mosquito house entry and rates of vector borne disease is sparse but some studies have indicated that wood smoke repels mosquitoes from homes [26], [27], [28]. In contrast, a study investigating malaria in the Ethiopian highlands found an increased risk of malaria when cooking took place inside the house [23]. However, in the Ethiopian study it is possible that cooking within the main house was a sign of lower socioeconomic status which is known to be linked with increased risk of malaria. In a recent systematic review Biran and others [5] concluded that smoke from domestic fires was unlikely to reduce mosquito feeding, but that burning repellent plants might be an effective way to reduce bites. They suggested reduced moisture content in the air as a hypothesis for the spatial repellent effect of wood smoke but the physiological effect of wood smoke on mosquitoes is an area where more research is needed.

Although these results from the Lao PDR suggest that a smoky kitchen might be advantageous in reducing rates of mosquito house entry, it would not be prudent to advocate the burning of biofuels inside houses given the well-established links between indoor air pollution and worsening of respiratory diseases [29], [30].

Previous studies of zoophilic vectors in Pakistan, Ethiopia and the Philippines [23], [31], [32] support our observation that cow ownership is associated with increased rates of anopheline house entry and this may be explained by zoophilic mosquitoes being more attracted to a house where cows emit high levels of carbon dioxide and attractive odours. Some of the mosquitoes attracted to the house by the cattle may ultimately be diverted inside the house to feed. The concept of zooprophylaxis, a process whereby non-host animals may be used to divert mosquito bites away from humans, thus lowering disease transmission risk [33], is therefore not recommended in this region at this time. Further studies analysing bloodmeals of mosquitoes captured inside houses could reveal whether mosquitoes attracted to cows subsequently feed on humans or whether they feed on cows before entering houses to rest. If the latter behaviour was demonstrated to be the case in the Lao PDR there could be a case for zooprophylaxis for malaria control in this area.

It is interesting that after adjusting for other risk factors, no significant association was found between cattle ownership and house entry by potential vectors of JE. Other studies have shown that *Cx. tritaeniorhynchus, Cx. vishnui* and *Cx. pseudovishnui* exhibit strong preferences for feeding on cattle [34]. A higher proportion of families living in wooden houses owned cattle, compared with families living in bamboo houses, although these differences were not statistically significant (results not presented here). The practice of keeping large animals below the house at night was generally more common in rice farming villages (34.6% of households) than in the resettlement area (16.7% of households) and if vectors exhibited zoophagic preferences this may help to explain the greater overall risk of putative JE vectors in houses in the rice farming area compared with the resettlement area.

Ideally this study would have been carried out in a larger sample size of houses, all located in the resettlement area, in order to limit geographical variation within the sample. However, traditional houses in the resettlement area were too few for this approach to be taken. In addition, within the resettlement area catch sizes of anopheline mosquitoes were too low for us to investigate risk factors for house entry by this group of mosquitoes. This study may have been underpowered to detect an effect of cattle ownership on rates of JE vector house entry. As described above, results of univariate analysis suggested that houses owning cows and buffaloes were at increased risk of putative JE vectors however, after including other predictors in the model this association was no longer significant. Difficulties with obtaining accurate data regarding the actual number of large animals kept by householders meant that this variable was treated as a binomial predictor. Knowledge of the actual number of animals kept by each household may have allowed a direct linear association between this risk factor and putative JE vector entry to be elucidated.

Despite geographical variation in mosquito densities between the two types of villages our results consistently show that improvements in house construction, such as building houses from wood rather than bamboo, can have a beneficial effect on reducing a person’s exposure to mosquitoes. With increasing socioeconomic development in the Lao PDR and the wider South-East Asian region it is hoped that housing quality will improve and that household-level exposure of people to potentially infective mosquito bites will be lowered. Where governments and commercial companies are implementing resettlement programs for local populations it is strongly recommended that full consideration of housing design be taken in to account. Wherever possible, houses should be built from high quality hardwood, much in line with the style of housing used in the NT2 resettlement programme.
